# Generalized Comedones, Acne, and Hidradenitis Suppurativa in a Patient with an *FGFR2* Missense Mutation

**DOI:** 10.3389/fmed.2017.00016

**Published:** 2017-02-28

**Authors:** Rebecca Higgins, Andrew Pink, Robert Hunger, Nikhil Yawalkar, Alexander A. Navarini

**Affiliations:** ^1^Department of Dermatology, University Hospital of Zurich, Zurich, Switzerland; ^2^King’s College, St John’s Institute of Dermatology, London, UK; ^3^Department of Dermatology, Inselspital, Bern University Hospital and University of Bern, Bern, Switzerland

**Keywords:** acne, comedones, fibroblast growth factor-receptor gene 2, hidradenitis suppurativa, whole exome sequencing

## Abstract

Mutations in the fibroblast growth factor-receptor gene 2 (*FGFR2*) gene have been implicated in numerous diseases, including nevus comedonicus (NC) and naevoid acne that have somatic missense mutations in *FGFR2* in the affected tissue. A patient presented in our department with unusual, innumerable large comedones throughout his back reminiscient of NC, as well as multifocal hidradenitis suppurativa and acne. Topical and systemic treatments were unsuccessful. Whole exome sequencing of blood-derived DNA detected a germline mutation in *FGFR2* that was predicted to be damaging. This could explain the multifocal and severe nature of the disease. We suggest screening other, phenotypically similar patients for *FGFR2* mutations. Our findings, once confirmed independently, could indicate that therapeutic modulation of FGFR signaling in the acne tetrad could be effective.

Hidradenitis suppurativa (HS) is an inflammatory skin disease that affects roughly 1% of Europeans. It is characterized by painful abscesses, boils, cysts, and malodorous, pus-filled lesions in intertriginous regions (1, 2). It can follow autosomal dominant inheritance in some families, and mutations have been reported (3) in the gamma-secretase genes Presenilin-1 (*PSEN1*), Presenilin Enhancer-2 (*PSENEN*), and Nicastrin (*NCSTN*) in a minority (<7%) of cases. The remaining cohort are presumed to be sporadic or driven by other, as yet unidentified mutations. Nevus comedonicus (NC) is a rare (prevalence 1:45,000–1:100,000) epidermal nevus comprising of a group of dilated hair follicle openings filled with plugs of brownish-black oxidized keratin (4). It is usually localized on the head and neck and was initially described as “localized acne” (5). If it occurs as a part of the nevus comedonicus syndrome (NCS) (6), it can be generalized and form linear streaks. HS-like lesions have been observed in NCS (7). In some cases of NC, a somatic mutation in fibroblast growth factor-receptor gene 2 (*FGFR2*) has been identified, namely the Ser252Trp missense mutation (5). FGFR2 is expressed in keratinocytes, hair follicles, and sebaceous glands and has been implicated to induce hypercornification and comedogenesis (8). Germline *FGFR2* mutations are also associated with acne, as seen in dominant Apert syndrome (9) (also comprising craniosynostosis, epiphyseal closure, and syndactyly) (10, 11). When occurring as a mosaic, the acne lesions follow Blaschko’s lines (12), the pattern of embryological cell development and proliferation. In a recent case, a postzygotic mosaicism was found in exon 4 of *FGFR2* (c.758C>G, p.Pro253Arg) in low copy number. A similar mosaicism was also found in two other patients in p.Ser252Trp of exon 4 (8).

A 49-year-old male construction worker presented with a 29-year history of multiple abscesses in his buttock, inguinal, and axillary regions (Figure 1) and a history of severe post-pubertal acne conglobate. His back was studded with innumerable large comedones not unlike those seen in NC, however rather more spread out (Figure 1A), and with significant scarring. His condition had proved resistant to treatment with topical and systemic antibiotics, dapsone, retinoids, including acitretin and isotretinoin, zinc supplements and PUVA. Treatment with infliximab did have an effect on the inflammatory components; however, it was minimal and ultimately unsuccessful. Surgical interventions included excision of the left axilla and shoulder region and a transposition flap and excision of a pilonidal sinus.

**Figure 1:**
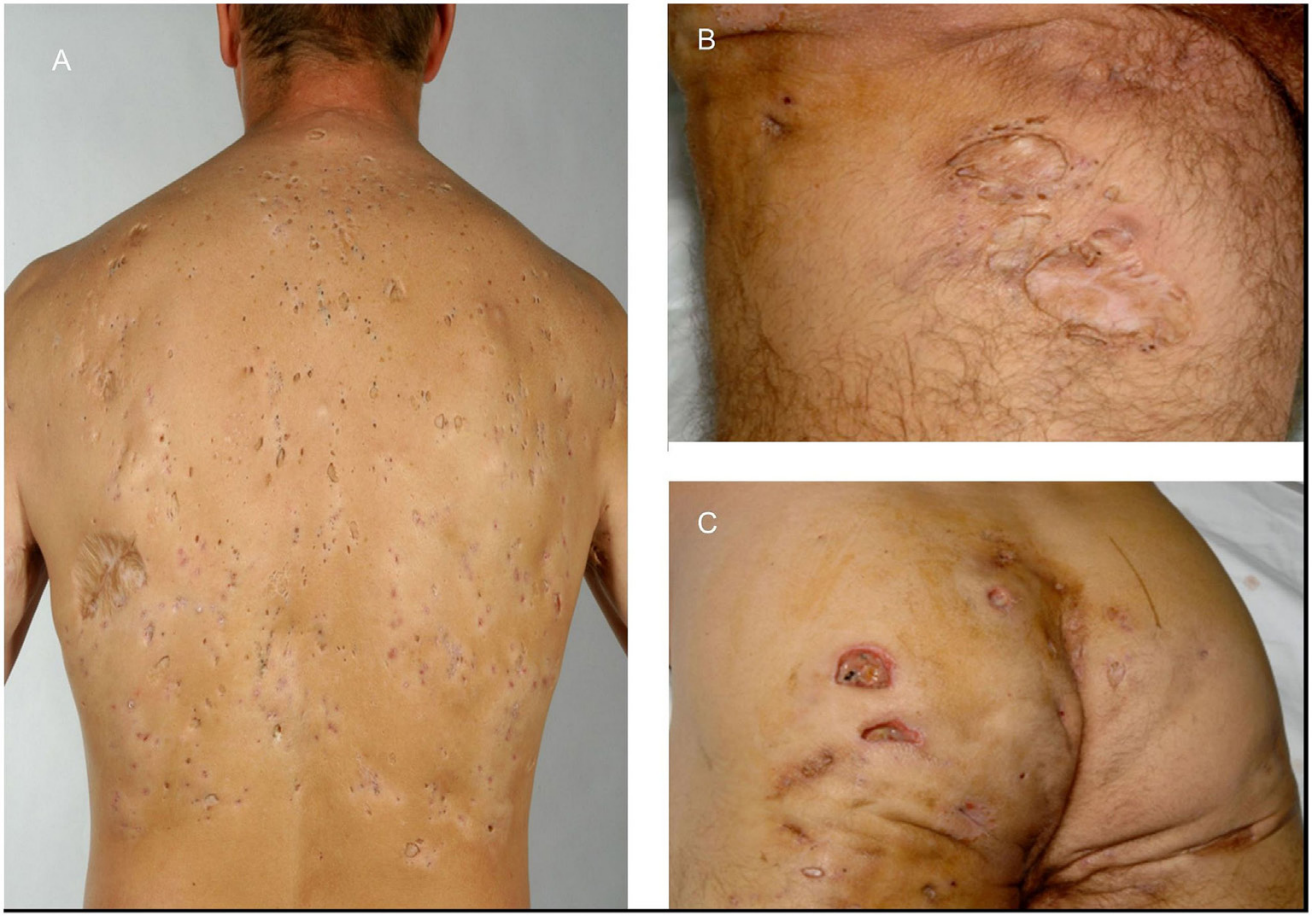
**Widespread abscesses present in 49-year-old male patient**. **(A)** Back of patient showing grouped comedones and hidradenitis suppurativa (HS) scars. **(B)** Inguinal scars. **(C)** Close-up of open HS lesions.

The patient is an orphan. It is known that several other people in his family including his father and brother had suffered from the same condition with different intensities; however, there is no contact between the patient and family. Requests to collect samples from family members were denied by the patient. Suspecting a potential genetic origin (13), we therefore performed whole exome sequencing on blood-derived DNA. A search for rare, deleterious mutations revealed that there was no such mutation present in any of the known HS-associated genes. However, a rare missense mutation in the *FGFR2* gene was found in exon 5 (c.G492C, p.K164N, see Table 1). This mutation is predicted (14) to have pathological consequence on the protein by several prediction algorithms, namely SIFT (“deleterious,” lowest score 0), PolyPhen (“probably damaging,” highest score 1), LoFtool (“probably damaging,” 0.00179), Condel (“deleterious,” 1.00), CADD (17.05), and SNPs&Go (73% probability). MutPred resulted in 63% probability for “damaging,” and predicted (15) molecular features of the mutation are loss of methylation at K164 (*P* = 0.0069), loss of ubiquitination at K164 (*P* = 0.0081), loss of solvent accessibility (*P* = 0.0371), and loss of MoRF binding (not significant, *P* = 0.0575) as well as gain of sheet (not significant, *P* = 0.1451).

**Table 1:** **Fibroblast growth factor-receptor gene 2 (FGFR2) mutation identified in the patient**.

Chr	Pos	Ref	Alt	Exon	Mutation	MAF in 1KG, EVS, ExAC
10	123310936	C	G	exon5	c.G492C, p.K164N	0.00
**SIFT**	**PolyPhen**	**Condel**	**LoFtool**	**SNPs&Go**	**MutPred**	**CADD**
deleterious	probably_damaging	deleterious (1.0)	probably damaging (0.00179)	Disease (0.73)	Disease (0.626)	17.1

## Discussion

In a patient with clinical features reminiscent of NCS, we found a new, rare heterozygous missense mutation in *FGFR2*, the gene for the keratinocyte growth factor (KGF) receptor. FGFR2, also known as CD332, was cloned in 1990 (16) and soon found to be the receptor for KGF. It has an extracellular part constructed of three immunoglobulin domains, a lipophilic transmembrane part, and a tyrosine kinase that extends in the cytoplasm. Fibroblast growth factors (FGF) can bind the extracellular domain and thus activate the tyrosine kinase. This leads to cell division and differentiation. The isoform found in ectodermal tissues such as the skin is the FGFR2IIIb, also known as the KGF receptor. It binds FGF-7 and 10, also known as KGF 1 and 2, as well as FGF-1, -3, -22. Keratinocyte growth factors are potent mitogens for many epithelial cell types but lack detectable activity on fibroblasts or endothelial cells.

Germline mutations in *FGFR2* underlie various craniosynostosis syndromes (Table 2), and somatic mutations in the same gene have been reported in NC (5) and nevoid acne (9). We report a germline mutation in *FGFR2* that may contribute to explaining a generalized, rather than mosaic, NC-like, acneiform phenotype with additional features of HS. As documented above, the disease proved highly treatment resistant, which is a common feature in NC. FGFR inhibitors, which may directly address the genetically driven pathogenesis, exist but have not been reported in this context.

**Table 2:** **Diseases caused by FGFR2 mutations**.

Location	Phenotype	Phenotype MIM number	Inheritance	Phenotype mapping key
10q26.13	Antley–Bixler syndrome without genital anomalies or disordered steroidogenesis	207410	AR	3
10q26.13	Apert syndrome	101200	AD	3
10q26.13	Beare-Stevenson cutis gyrata syndrome	123790	AD	3
10q26.13	Bent bone dysplasia syndrome	614592	AD	3
10q26.13	Craniofacial-skeletal-dermatologic dysplasia	101600	AD	3
10q26.13	Craniosynostosis, non-specific			3
10q26.13	Crouzon syndrome	123500	AD	3
10q26.13	Gastric cancer, somatic	613659		3
10q26.13	Jackson–Weiss syndrome	123150	AD	3
10q26.13	LADD syndrome	149730	AD	3
10q26.13	Pfeiffer syndrome	101600	AD	3
10q26.13	Saethre–Chotzen syndrome	101400	AD	3
10q26.13	Scaphocephaly and Axenfeld-Rieger anomaly		NA	3
10q26.13	Scaphocephaly, maxillary retrusion, and mental retardation	609579	NA	3
10q26.13	Nevus comedonicus	–	Somatic	none

As the patient is a foundling, we could not determine the presence of the mutation in the rest of the family and were unable to obtain material for tissue expression studies. All available algorithms unanimously predict impaired protein function as a consequence of this mutation but follow-up studies, identifying further similar cases, are warranted to confirm this genotype–phenotype correlation. HS is a phenotypically heterogeneous condition, and ongoing genetic investigation represents one important way of classifying subtypes and related conditions.

In summary, we have identified a germline mutation in *FGFR2* in a patient exhibiting features of generalized comedones and HS. Ongoing screening and functional interrogation of this group has the potential to further characterize the related phenotype and identify novel therapeutic targets.

Written informed consent was obtained from the patient to perform genetic analysis on the blood and to publish the resulting study.

## Ethics Statement

The presented findings were achieved in the context of clinical evaluation of this patient. They are presented with the patient’s written permission.

## Author Contributions

AN supervised the study, participated in data generation and analysis, and wrote the paper. R Higgins generated and analysed data and wrote the paper. R Hunger and NY contributed samples and clinical data.

## Conflict of Interest Statement

The authors declare that the research was conducted in the absence of any commercial or financial relationships that could be construed as a potential conflict of interest.

## References

[1] Pink AE Simpson MA Desai N Dafou D Hills A Mortimer P Mutations in the γ-secretase genes NCSTN, PSENEN, and PSEN1 underlie rare forms of hidradenitis suppurativa (acne inversa). J Invest Dermatol (2012) 132:2459–61. 10.1038/jid.2012.162 22622421

[2] Pink AE Simpson MA Desai N Trembath RC Barker JNW. γ-Secretase mutations in hidradenitis suppurativa: new insights into disease pathogenesis. J Invest Dermatol (2013) 133:601–7. 10.1038/jid.2012.372 23096707

[3] Wang B Yang W Wen W Sun J Su B Liu B Gamma-secretase gene mutations in familial acne inversa. Science (2010) 330:1065. 10.1126/science.1196284 20929727

[4] Kofmann S Ein Fall von seltener Localisation und Verbreitung von Comedonen. Arch Dermatol (1895) 32:177–8.

[5] Munro CS Wilkie AO Epidermal mosaicism producing localised acne: somatic mutation in FGFR2. Lancet (1998) 352:704–5. 9728990 10.1016/S0140-6736(05)60820-3

[6] Engber PB. The nevus comedonicus syndrome: a case report with emphasis on associated internal manifestations. Int J Dermatol (1978) 17:745–9. 730460 10.1111/ijd.1978.17.9.745

[7] Qian G Liu T Zhou C Zhang Y Naevus comedonicus syndrome complicated by hidradenitis suppurativa-like lesions responding to acitretin treatment. Acta Derm Venereol (2015) 95:992–3. 10.2340/00015555-2089 25758459

[8] Melnik B. Role of FGFR2-signaling in the pathogenesis of acne. Dermatoendocrinol (2009) 1:141–56. 20436882 10.4161/derm.1.3.8474 PMC2835907

[9] Campanati A Marconi B Penna L Paolinelli M Offidani A. Pronounced and early acne in Apert’s syndrome: a case successfully treated with oral isotretinoin. Eur J Dermatol (2002) 12:496–8. 12370145

[10] Moloney DM Slaney SR Oldridge M Wall SA Sahlin P Stenman G Exclusive paternal origin of new mutations in Apert syndrome. Nat Genet (1996) 13:48–53. 8673103 10.1038/ng0596-48

[11] Downs AM Condon CA Tan R. Isotretinoin therapy for antibiotic-refractory acne in Apert’s syndrome. Clin Exp Dermatol (1999) 24:461–3. 10606949 10.1046/j.1365-2230.1999.00533.x

[12] Kiritsi D Lorente AI Happle R Bernabeu Wittel J Has C Blaschko line acne on pre-existent hypomelanosis reflecting a mosaic FGFR2 mutation. Br J Dermatol (2015) 172:1125–7. 10.1111/bjd.13491 25350236

[13] Fitzsimmons JS Guilbert PR Fitzsimmons EM. Evidence of genetic factors in hidradenitis suppurativa. Br J Dermatol (1985) 113:1–8. 4015966 10.1111/j.1365-2133.1985.tb02037.x

[14] McLaren W Pritchard B Rios D Chen Y Flicek P Cunningham F. Deriving the consequences of genomic variants with the Ensembl API and SNP Effect Predictor. Bioinformatics (2010) 26:2069–70. 10.1093/bioinformatics/btq330 20562413 PMC2916720

[15] Li B Krishnan VG Mort ME Xin F Kamati KK Cooper DN Automated inference of molecular mechanisms of disease from amino acid substitutions. Bioinformatics (2009) 25:2744–50. 10.1093/bioinformatics/btp528 19734154 PMC3140805

[16] Houssaint E Blanquet PR Champion-Arnaud P Gesnel MC Torriglia A Courtois Y Related fibroblast growth factor receptor genes exist in the human genome. Proc Natl Acad Sci U S A (1990) 87:8180–4. 2172978 10.1073/pnas.87.20.8180 PMC54916

